# In Vitro Models of Triple-Negative Breast Cancer of Sub-Saharan African Ancestry Suggest *BLM*-Centred DNA Damage Repair Dependency and Pevonedistat Sensitivity

**DOI:** 10.3390/ijms27219980

**Published:** 2026-11-07

**Authors:** Ricardo J. Pinto, Ana C. Magalhães, Clara M. Bento, Fernanda Rosário, Lúcio Lara Santos, Emanuel Gonçalves, Luisa Pereira

**Affiliations:** 1 i3S—Instituto de Investigação e Inovação em Saúde, Universidade do Porto, 4200-135 Porto, Portugal; rjpinto@i3s.up.pt (R.J.P.); acmagalhaes@i3s.up.pt (A.C.M.); clara.bento@i3s.up.pt (C.M.B.);; 2 IPATIMUP—Instituto de Patologia e Imunologia Molecular da Universidade do Porto, 4200-135 Porto, Portugal; 3 ICBAS—Instituto de Ciências Biomédicas Abel Salazar, Universidade do Porto, 4050-313 Porto, Portugal; 4 Research Center of IPO-Porto (CI-IPOP)/RISE@CI-IPOP (Health Research Network), Portuguese Oncology Institute of Porto (IPO-Porto)/Porto Comprehensive Cancer Center (P.CCC) Raquel Seruca, 4200-162 Porto, Portugal; lucio.santos@ipoporto.min-saude.pt; 5 FP-I3ID—Instituto de Investigação, Inovação e Desenvolvimento da Fundação Fernando Pessoa, University Fernando Pessoa, 4200-253 Porto, Portugal; 6 Department of Surgical Oncology, Portuguese Oncology Institute of Porto, 4200-162 Porto, Portugal; 7 School of Medicine and Biomedical Sciences, University Fernando Pessoa, 4420-096 Gondomar, Portugal; 8 Instituto Superior Técnico (IST), Universidade de Lisboa, 1049-001 Lisboa, Portugal; emanuel.v.goncalves@tecnico.ulisboa.pt; 9 Instituto de Engenharia de Sistemas e Computadores—Investigação e Desenvolvimento (INESC-ID), 1000-029 Lisboa, Portugal

**Keywords:** triple-negative breast cancer, cancer cell lines, sub-Saharan African ancestry, gene essentiality, drug sensitivity

## Abstract

Triple-negative breast cancer (TNBC) is more prevalent in women of sub-Saharan African (SSA) than European (EUR) ancestry. Despite ancestry-related heterogeneity remaining neglected in cancer research, its evaluation can reveal useful insights on essential pathways underlying complex diseases. High-throughput data (DepMap and GDSC) were mined by performing differential analyses (gene essentiality and drug sensitivity, respectively) between SSA and EUR TNBC cell lines. *BLM* and interacting genes (*RMI1*, *RMI2*, *TOP3A*) showed greater essentiality in SSA than in EUR TNBC cell lines. SSA TNBC patients and cancer cell lines tended to have a lower median *BLM* expression than EUR counterparts, and its expression was positively correlated with structural mutations in tumour cells. Concordantly, *BLM* expression was higher in TNBC tumour cells than in normal cells, suggesting activation due to accumulation of mutations, particularly in cancers with a high burden of structural mutations. SSA TNBC cell lines were also more sensitive to the drug Pevonedistat. Transcriptomic analyses revealed that in the drug-exposed condition, DNA damage repair and helicase-related pathways were significantly downregulated, in several of which *BLM* is a significant core gene. Our findings support incorporating ancestry into oncobiology in vitro modelling and conducting further studies to assess Pevonedistat as a potential TNBC treatment.

## 1. Introduction

The abundance of omics data for the Cancer Cell Line Encyclopedia (CCLE) renders it extensively mined by various research groups, who have been revealing important insights into oncology mechanisms and the diverse factors influencing them [1,2]. In fact, the CCLE is a remarkable tool enabling the functional investigation of several hypotheses, especially when coupled with the powerful method of genome-wide CRISPR-Cas9 [3,4]. Pooled CRISPR-Cas9 screens employing genome-scale libraries of single-guide RNAs are being performed on the CCLE [5], allowing the identification of essential genes, that is, genes required for the survival and proliferation of cancer cells. A practical consequence of this evidence is that it can be prioritized to develop new cancer therapeutic targets, as illustrated in Behan et al. 2019 [6]. This later work identified the Werner syndrome ATP-dependent helicase (*WRN*) as one of the most promising dependencies, suggesting that it should be considered as a synthetic lethal target in tumours from multiple cancer types with microsatellite instability (MSI). CCLE has also been used to conduct high-throughput drug screening and relate drug sensitivities with cancer-driven alterations [7]. The authors mapped cancer-driven alterations (identified in 11,289 tumours from 29 tissues) onto 1001 molecularly annotated human cancer cell lines and correlated them with sensitivity to 265 drugs. This demonstrated that these CCLE datasets are rich resources to link genotypes with cellular phenotypes and to identify therapeutic options for selected cancer sub-populations.

A factor that is beginning to attract interest in the field of oncology mechanisms is ancestry [8,9], as several genomic profile studies, such as in prostate [10,11,12] and breast [13,14,15] cancers, have been providing evidence that genomic differences in cancers exist among ancestries. The major source of the omics data comes from The Cancer Genome Atlas (TCGA), but it is not the only one. Yuan et al. [16] detected that, compared with European (EUR) ancestry, African Americans exhibited higher levels of chromosomal instability (CIN) in breast, head and neck, and endometrial cancers, and the opposite tendency for kidney cancers; increased *TP53* mutation frequency and amplification of the cyclin E1 (*CCNE1*) gene in the cancer types showing higher CIN; lower frequencies of genomic alterations affecting genes in the PI3K pathway across cancers. Carrot-Zhang et al. [17] evaluated ancestry effects on mutation rates, DNA methylation, and mRNA and miRNA expression profiles in almost the same set of TCGA patients, and concluded that when ancestry is taken into account, it is responsible for differences spanning all molecular features and hundreds of genes, usually tissue-specific and not cancer-specific; however there were cancer-specific differences such as increased *FBXW7* (involved in ubiquitination; the protein coded by this gene binds directly to the oncoprotein cyclin E, probably targeting it for ubiquitin-mediated degradation [18]) mutations across cancer types and decreased *VHL* and *PBRM1* mutations in renal cancer in African patients; significant correlation between African and aneuploidy in pan-cancer analyses when not controlling for cancer subtype; methylation data highlighted *FAAP20* (part of Fanconi anemia nuclear complex, involved in inter-strand cross-link repair and translesion synthesis) in African samples, as highly methylated and hence lower expression; when integrating ancestry, methylation and expression data, BC showed significant differences in “key” regulatory nodes, with *ATM*, *SP1* and *MAPK14* remaining significant in subtype-adjusted analyses; and when assessing cancer pathways and driver genes, significantly enriched pathways included DNA repair, HIPPO, RTK-RAS, p53, NRF2, and Notch pathways in the breast cancer (BC) African-European comparison. All these results reinforce the need to improve omics tumour cataloguing of non-EUR ancestry and provide interesting insights into possible ancestry-associated differences in response to diverse treatments. In summary, for the sub-Saharan African (SSA) ancestry, a major involvement of cell cycle deregulation/chromosomal instability/DNA repair seems to take place in several cancer types.

In this work, we mined the various omics of CCLE and TCGA consortia to (1) investigate whether we could link the distinct SSA-EUR cancer features with differential gene dependencies; and (2) identify therapeutic options for the SSA ancestry. We decided to focus on triple-negative breast cancer (TNBC) as this subtype of BC has a higher incidence in women of SSA ancestry than in EUR ancestry, and it is usually associated with a poor prognosis when compared with luminal and HER2-enriched subtypes [19,20,21,22]. Lately, biomarker-directed treatments (PARP inhibitors for *BRCA* mutations) and antibody-drug conjugates (anti-PD-L1 agents) are offering some promise for TNBC treatment [23]. There are five SSA TNBC cell lines in the CCLE and 25 SSA TNBC patients in the TCGA (inferred from ancestry affiliations provided by [17,24], respectively), just enough to provide us with very interesting evidence and appeal for action to establish more in vitro models representative of the SSA ancestry [25].

## 2. Results

### 2.1. BLM as a Putative Essential Gene in TNBC for SSA Ancestry

**Figure 1:**
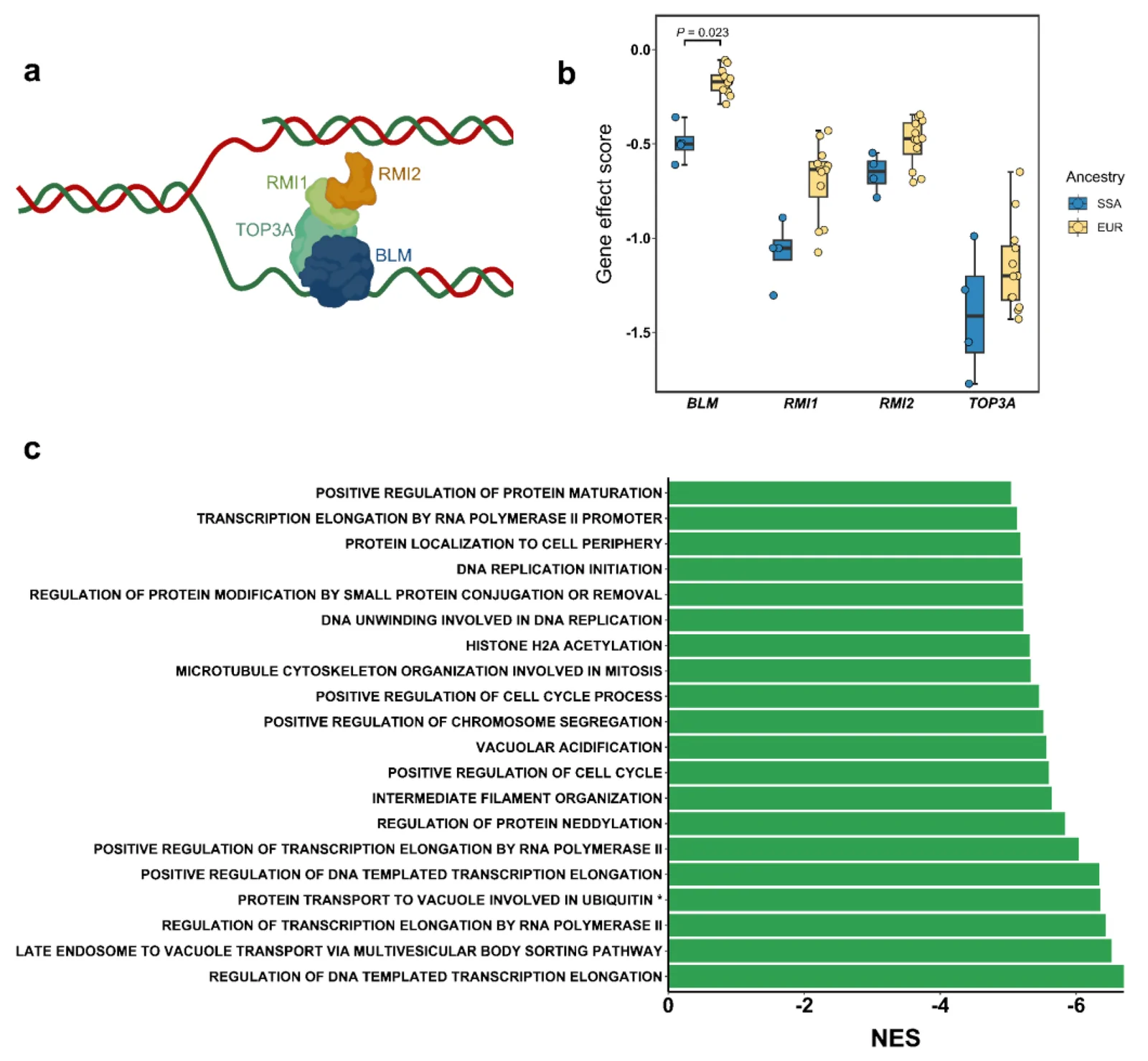
(**a**) BLM interaction with partners within HR DNA repair. (**b**) Significant hit for the *BLM* gene in SSA ancestry, and the same tendency (not statistically significant) shared with partners from (a) (SSA cell lines = 4; EUR cell lines = 12). (**c**) Enrichment results (GO-BP) for differential essentiality between SSA and EUR groups. * Protein transport to vacuole involved in ubiquitin-dependent protein catabolic process via the multivesicular body sorting pathway.

The analysis to compare the differential gene essentiality between SSA and EUR TNBC cell lines identified a significant hit for the gene BLM RecQ like helicase (*BLM*), after adjustment for multiple comparisons, being stronger in SSA than in EUR ancestry (gene essentiality scores: SSA = −0.50; EUR = −0.17; adjusted *p*-value = 0.023; log2-fold changes [logFC] = −0.32; 95% confidence interval [CI] = [−0.42, −0.23]; negative scores suggest the gene is essential for the survival of a specific cell line). Negative values are indicative of a detrimental impact of a knockout gene on cell survival. Specifically, non-essential genes cluster around a score of 0, whereas a score of −1 represents the median dependency level of established pan-essential genes Figure 1a; Tables S1 and S2. To assess the robustness of the *BLM* differential essentiality signal, a leave-one-out (LOO) sensitivity analysis was performed, confirming logFC values consistently negative in the range −0.285 to −0.369 (Table S3). Obviously, removing one SSA cell line led to non-significant adjusted *p*-values in three of four cases, due to the low sample size in this class.

*BLM* encodes the Bloom syndrome helicase, a pivotal member of the RecQ family of DNA helicases [26]. These highly conserved proteins play a critical role in the intricate process of DNA unwinding during repair and replication processes, being essential for maintaining genome integrity [27]. Particularly, *BLM* has been shown to contribute to genome fidelity mainly through its involvement in the repair of DNA double-strand breaks or the restoration of stalled replication forks by homologous recombination (HR) [28]. To perform these functions, the assembly of a complex denominated BLM syndrome complex, also known as BLM dissolvasome or BTRR, is required. This complex consists of BLM, DNA topoisomerase IIIa (TOP3A), and RecQ-mediated instability proteins 1 and 2 (RMI1 and RMI2; Figure 1a) [29]. Although not statistically significant in the linear model analysis after correction for multiple testing, we observed a consistent trend of higher gene essentiality for all partners of BLM in SSA in comparison to EUR cell lines, within the HR DNA repair (*RMI1*: SSA = −1.05, EUR = −0.64; *RMI2*: SSA = −0.65, EUR = −0.47; *TOP3A*: SSA = −1.41, EUR = −1.20; Figure 1a,b). Due to the low number of available TNBC cell lines, the statistical power of the linear model is quite limited. We then computed Single Sample Gene Set Enrichment Analysis (ssGSEA) on the differential essentiality between SSA and EUR groups for all genes, sorted in descending order of limma logFC estimates of essentiality, and identified significantly enriched results, promoting dependency in SSA TNBC cell lines, for 114 gene sets in the Gene Ontology (GO)–Biological Processes (BP) class, 42 in GO–Cellular Components (CC), and 16 in GO–Molecular Function (MF) (Tables S4–S6). Pathways related to DNA repair and replication feature prominently among the top hits for the SSA ancestry (Figure 1c), as well as pathways related to ubiquitination and neddylation, which are post-translational modifications (PTMs) of proteins, regulating their stability, activity and subcellular localization [30]. It was demonstrated that BLM protein is post-translationally modified, such as by small ubiquitin-related modifiers (SUMOs), regulating the trafficking of the BLM helicase to DNA damage-induced foci [31,32]. The enrichment in these pathways in SSA ancestry further strengthens the signal previously reported for *BLM* dependency in SSA background and underscores its critical biological role.

To validate the linear methodology implemented in this analysis and dismiss the possibility of a spurious association for *BLM*, we further constructed a linear model using the Genomics of Drug Sensitivity in Cancer (GDSC) project drug sensitivity data in relation to *B-Raf proto-oncogene* (*BRAF*) mutational status (*BRAF* mutated vs. *BRAF* wild-type). If the top hits are known drugs acting on this gene, this is an indication that the linear methodology is powerful in identifying the significant candidates. The results of this validation (Table S7) show that the top 13 associations target the MAPK/ERK pathway (also known as the Ras-Raf-MEK-ERK pathway), three of which seem to act directly on *BRAF*, while the others have other putative targets in that pathway. These findings provide additional support for the validity of the differential essentiality approach used in this study, and that this analytical framework could recover a known pharmacogenomic association.

In order to provide a reference value for the strength of *BLM* essentiality in SSA TNBC cell lines, we evaluated the dependency of cancer cell lines of all origins on the *BRAF* by comparing the presence and absence of the *BRAF*^V600E^ gain-of-function mutation, which mimics a phospho-activating state of the protein [33]. We observed that the difference in essentiality between the *BRAF*^V600E^ and wild-type *BRAF* groups was comparable in magnitude to the difference observed between ancestries for *BLM* essentiality (*BRAF*^V600E^ = −0.58, wild-type *BRAF* = −0.07; Figure S1).

### 2.2. BLM Gene Expression in TNBC Patient Samples and Cell Lines

**Figure 2:**
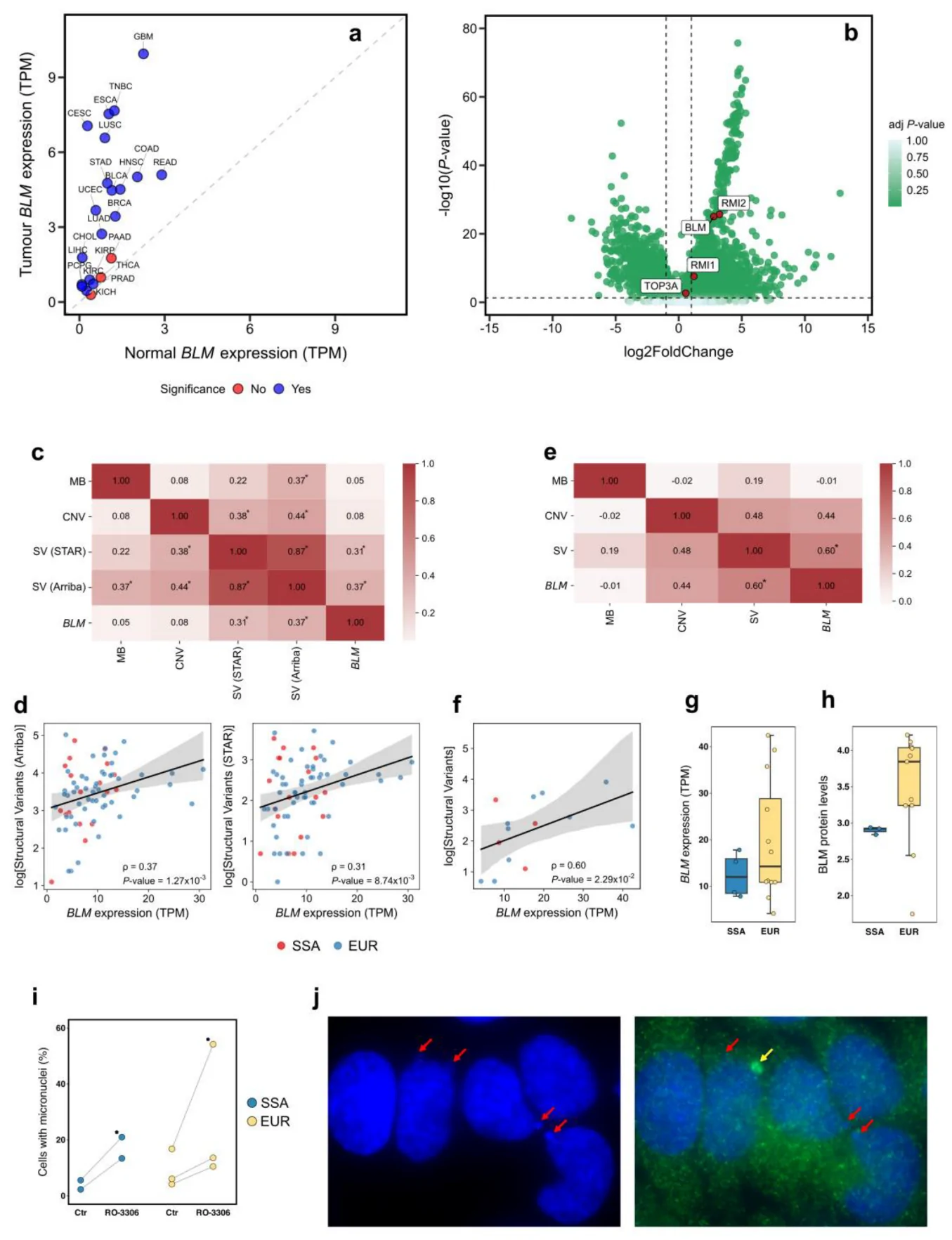
*BLM* gene expression in cancer context. (**a**) Comparison of *BLM* gene expression (transcripts per million [TPM]) between tumour and normal tissues in TCGA cohorts (cohorts with less than three normal samples were excluded). (**b**) Volcano plot for differential gene expression between TCGA TNBC tumour and normal tissues. (**c**,**e**) Heatmaps for the correlations between *BLM* gene expression and somatic mutational profiles in TNBC TCGA and cancer cell lines, respectively (SSA TCGA cases = 18; EUR TCGA cases = 58; SSA cell lines = 4; EUR cell lines = 10; * statistically significant correlations). (**d**,**f**) Scattered dot plots for the significant correlations between *BLM* gene expression and SVs in TNBC TCGA and cancer cell lines, respectively (SSA TCGA cases = 18; EUR TCGA cases = 58; SSA cell lines = 4; EUR cell lines = 10). (**g**) Boxplot for the *BLM* mRNA expression in SSA and EUR TNBC cell lines (data from CMP; SSA cell lines = 4; EUR cell lines = 16). (**h**) Boxplot for the BLM protein expression in SSA and EUR TNBC cell lines (data from CMP; SSA cell lines = 3; EUR cell lines = 15). (**i**) Percentage of cells with micronuclei in control and when arrested at the G2/M boundary (RO-3306) in SSA and EUR TNBC cell lines (*n* = 2 vs. 3, respectively). * cancer cell lines with probable no expression of BLM protein and the lowest *BLM* mRNA expression (data from CMP). (**j**) Microscopic images (HCC1806 cell line, control group) evidencing micronuclei (left panel, red arrows) and whether they are cGAS-positive (right panel, yellow arrows) or negative (right panel, red arrows). DAPI (blue) indicates nuclei and micronuclei, while Alexa488 (green) indicates cGAS staining. Abbreviations: BLCA: bladder urothelial carcinoma; BRCA: breast invasive carcinoma; CESC: cervical squamous cell carcinoma and endocervical adenocarcinoma; CHOL: cholangiocarcinoma; CNV: copy number variant; COAD: colon adenocarcinoma; ESCA: oesophageal carcinoma; GBM: glioblastoma multiforme; HNSC: head and neck squamous cell carcinoma; KICH: kidney chromophobe; KIRC: kidney renal clear cell carcinoma; KIRP: kidney renal papillary cell carcinoma; LIHC: liver hepatocellular carcinoma; LUAD: lung adenocarcinoma; LUSC: lung squamous cell carcinoma; MB: mutational burden; PAAD: pancreatic adenocarcinoma; PCPG: pheochromocytoma and paraganglioma; PRAD: prostate adenocarcinoma; READ: rectum adenocarcinoma; STAD: stomach adenocarcinoma; SV: structural variant; THCA: thyroid carcinoma; TNBC: triple-negative breast cancer; UCEC: uterine corpus endometrial carcinoma.

A previous study on 2000 BC tumour samples revealed *BLM* mRNA overexpression and its significant association with high histologic grade, larger tumour size, estrogen receptor (ER), and progesterone receptor (PR) status [34]. We confirmed that *BLM* gene expression is significantly increased in BC and TNBC tumour tissues in relation to normal tissues, as well as for most other cancer types in TCGA (Figure 2a,b). The highest differences are observed for glioblastoma and TNBC. It is impossible to verify this for hematologic malignancies, as there is no data for normal cells, but looking only at the tumour data (Figure S2—Figure S2a), the hematologic malignancies occupy the top positions of *BLM* expression. This pattern is almost parallel in the cancer cell lines (Figure S2—Figure S2b).

As *BLM* plays a role in DNA repair, we then investigated the correlation between all types of somatic alterations in both TCGA and cancer cell line TNBC cohorts and the expression of *BLM*. We observed a significant positive correlation between *BLM* mRNA levels and the number of structural variants (SVs) per sample, although the correlation is not very high (for both algorithms to infer SVs; Arriba: ρ = 0.37, *p*-value = 1.27 × 10^−3^; STAR: ρ = 0.31, *p*-value = 8.74 × 10^−3^; Figure 2c,d). Cases having high *BLM* expression levels seem to have a high number of SVs, while cases with lower *BLM* expression have variable rates of SVs; this heterogeneity is most probably due to the action of diverse mechanisms of DNA repair besides *BLM*-driven ones. The same significant correlation was observed in the TNBC cell lines (ρ = 0.60, *p*-value = 2.29 × 10^−2^; Figure 2e,f). The rho correlation coefficient is considerably higher for the cancer cell lines, probably due to the more permissive lab conditions for extreme *BLM* mRNA values. In the cancer cell lines, we checked for *BRCA1*/*BRCA2* mutations, as these genes are also involved in DNA damage repair (Table S8). Only four EUR lines (out of 12 EUR and 4 SSA) had possible cancer-driving mutations in *BRCA1*/*BRCA2*, but had heterogeneous levels of SVs (high, intermediate and low). These calculations were done without considering the ancestry value, as the number of samples with information for all features would be very limiting for these analyses. From the analysis of Figure 2d,f, SSA TNBC patients and cell lines generally exhibit lower mean values than the EUR counterparts (SSA = 7.34 and EUR = 10.10 for patients; SSA = 12.41 and EUR = 18.64 for cancer cell lines); mostly, no extremely high *BLM* mRNA expression values were observed in the SSA cohorts. Focusing only on the cancer cell lines, regardless of whether they were characterized for SVs (Figure 2g,h), it is possible to confirm the trend toward lower *BLM* mRNA and protein expression in SSA compared with EUR (Table S9).

Given the role of *BLM* in DNA repair, we evaluated micronuclei frequency in TNBC cell lines in vitro, as both micronuclei and SVs are established hallmarks of CIN [35]. Because micronuclei arise following anaphase, we assessed their formation under basal replication conditions as well as after G2/M boundary arrest induced by RO-3306. First, we checked the identity (through Short Tandem Repeats [STR] profiling; Table S10) and ancestry affiliation (through one million variants; Table S11) of all the TNBC cell lines used in our experiments, to avoid any mismatch during their acquisition and quarantine. Based on BLM protein data from Cell Model Passports (CMP) (Data Independent Acquisition [DIA]-Mass Spectrometry [MS]), we selected two SSA cell lines, one with low expression and one with no detection of BLM protein, while for EUR, we considered two with low expression and one with no detection of BLM protein. Although caution is required when interpreting missing values in MS data [36], BLM protein was detected in all other TNBC cell lines included in this study, except for these two cell lines (indicated with an asterisk in Figure 2i). As can be observed in the figure, both SSA and EUR cell lines in which BLM protein was not detected in the referenced proteomic dataset display a higher basal micronuclei frequency within their ancestry background, and have a higher increase in their frequency when stopping the cell cycle in G2/M (15 percentage points vs. 11 in SSA; 37 percentage points vs. the mean of seven in EUR). These micronuclei can induce an innate immune response, identified as cGAS^+^ micronuclei (Figure 2j), in a proportion of 6–34% of the total micronuclei (highest value for the EUR cell line in which BLM protein was not detected in the referenced proteomic dataset; Table S12).

### 2.3. Differential Sensitivity to Drugs Between SSA and EUR TNBC Cell Lines

**Figure 3:**
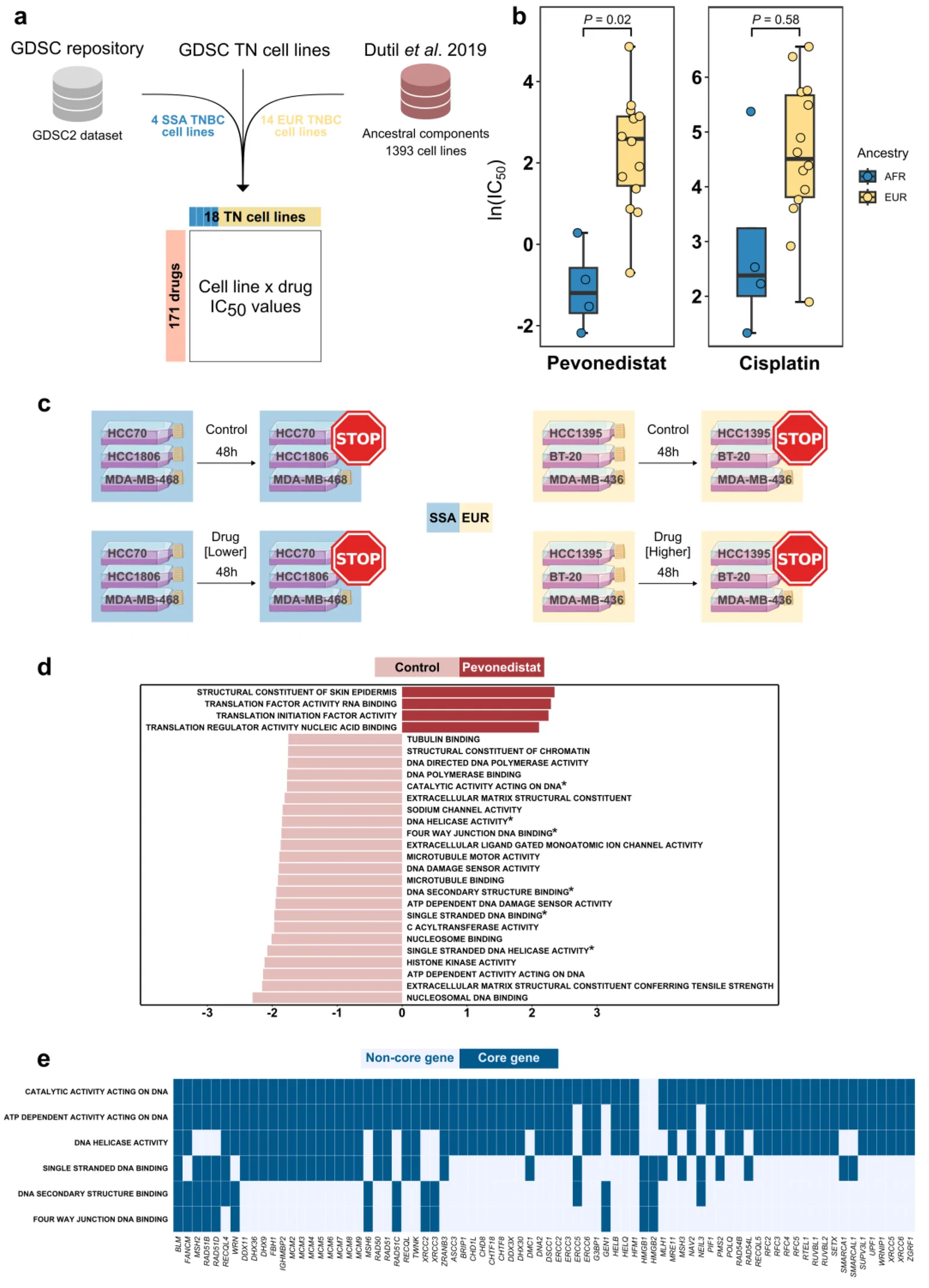
Evaluation of differential drug sensitivity between SSA and EUR TNBC cell lines. (**a**) Rationale for the GDSC2 evaluation of differential drug sensitivity between SSA (HCC1806, HCC70 and MDA-MB-468) and EUR (BT-20, HCC1395, MDA-MB-436) TNBC cell lines [24]. (**b**) Box plot of cell lines’ IC_50_ (μM; log scale) values for the only drug (Pevonedistat) in GDSC2 for which SSA and EUR ancestries have significant differential sensitivity and cisplatin (the *p*-value was adjusted for multiple testing with all drugs; SSA cell lines = 4; EUR cell lines = 14). (**c**) In vitro test of Pevonedistat in three SSA and EUR TNBC cell lines, at the respective median GI_50_ concentrations for each ancestral group ([Higher] 78.98 µM for EUR; [Lower] 0.72 µM for SSA). (**d**) Significant enrichment results (GO-MF) for the RNA-Seq data on the in vitro test represented in C, for both ancestries together; * pathways where *BLM* is a core gene. (**e**) The other core genes together with *BLM*.

Mining the GDSC2 dataset (Figure 3a) to evaluate TNBC ancestry influence on drug sensitivity (four SSA versus 14 EUR), the SSA cell lines were found to be significantly more sensitive to the drug Pevonedistat than their EUR counterparts (Figure 3b). The difference in the higher sensitivity of SSA vs. EUR cell lines to Pevonedistat’s effect (non-adjusted *p*-value = 8.96 × 10^−5^; adjusted *p*-value = 0.02; Table S13) surpassed that of cisplatin (non-adjusted *p*-value = 0.03; adjusted *p*-value = 0.58; Table S13), a drug belonging to the platinum-based agents, a group of chemotherapeutics already implemented in the treatment of TNBC patients (Figure 3b) [37]. Similar to the essentiality analysis, a LOO sensitivity analysis was also performed for the in silico differential drug sensitivity analysis. The estimated effect remained consistently negative across the LOO analyses, with logFC values ranging from −2.99 to −3.81 (Table S14). Pevonedistat, also known as MLN4924, is a small molecule that inhibits the Nedd8 activating enzyme (NAE), which may result in the inhibition of tumour cell proliferation and survival (potential antineoplastic activity [38,39]). NAE activates Nedd8 (neural precursor cell expressed, developmentally downregulated 8), a ubiquitin-like protein that modifies cellular targets to mark them for degradation. This result relates fittingly to the enrichment detected for the essentiality data, indicating that SSA cell lines were more dependent on ubiquitination and neddylation pathways, as reported in Section 2.1 (Figure 1c).

Given this evidence for Pevonedistat, we decided to conduct further in vitro testing of this drug in three SSA versus three EUR TNBC cell lines, to characterize the transcriptome landscape. First, we verified the higher sensitivity of SSA versus EUR to this drug and obtained the mean GI_50_ values for each ancestry in our assay (Figures S3 and S4; Table S15): 0.72 µM for SSA (described as [Lower]) and 78.98 µM for EUR (described as [Higher]). Then we conducted the test for each ancestry at the respective GI_50_ value to guarantee we retained sufficient viable cells for RNA extraction. Applying the higher concentration to both groups would have resulted in substantial cell loss in the SSA cell lines, potentially compromising RNA recovery, whereas using the lower concentration for both groups was expected to induce a weaker molecular response in the EUR cell lines (as illustrated in Figure 3c). In fact, growth inhibition measured at the time of cell collection for transcriptomic analysis confirmed that the selected concentrations resulted, on average, in approximately 50% inhibition of cell growth in both ancestry groups (SSA = 58.2%; EUR = 55.5%; Table S16). Enrichment analyses on the transcriptome data of the drug test (joining the two ancestries) (Figure 3d; Table S17) show a downregulation of *BLM*-centred DNA damage repair pathways in the Pevonedistat-exposed group when compared to the control condition. These pathways include “single-stranded DNA helicase activity”, “single-stranded DNA binding”, “DNA secondary structure binding”, “four-way junction DNA binding”, “DNA helicase activity” and “catalytic activity acting on DNA”. When considering the analysis stratified by ancestral group, the results follow the same trend (Figure S5; Tables S18 and S19). *BLM* and other related genes (Figure 3e) are core genes in these significantly altered pathways, although their individual decrease in expression in the presence of the drug is not statistically significant after multiple-testing correction, except for *MLH1*. Other related genes are other helicases (*MCM2-9* [40]), *BLM* interacting partners involved in DNA repair (*FANCM* [41], *MSH2* [42], *RAD51B* [43] and *RAD51D* [44]), and other members of the RecQ family of DNA helicases (*WRN*, *RECQL4, RECQL*).

## 3. Discussion

Whole-genome sequencing of a few hundred SSA individuals has revealed 20% greater genetic diversity compared to other ancestries [45,46,47,48], testifying that many novel variants remain undiscovered in the diverse ethnic groups from this vast continent [49,50]. The few SSA TNBC-available cancer cell lines were a good departure point to begin addressing the topic of ancestry influence, even if they are derived from AA individuals, which are a subset of the SSA genetic diversity. Because AA are an admixed group, requiring the SSA component to reach the mean ancestry estimate reported by Patin et al. (~70% SSA, ~30% European/Native American) ensured that the lines retained as SSA proxies carry a predominant SSA genetic contribution [51]. Being able to conduct this study in TNBC is particularly relevant, as this cancer subtype is known for its aggressive nature, poor prognosis, and disproportionately higher incidence in women of SSA descent [19,20,21,22].

The statistical analysis conducted in this study, based on CRISPR-Cas9 knockout screens of all protein-coding genes across the CCLE panel available through DepMap, identified *BLM* as displaying a significantly increased essentiality in SSA TNBC cell lines when compared to their EUR counterparts. Germline mutations in this gene are associated with Bloom syndrome (BS), a rare autosomal recessive monogenic disorder characterized by growth deficiency, infertility, sun-induced skin sensitivity (rash and pigmentation alterations), and increased genetic instability and predisposition to diabetes and cancer [52,53]. Together with *WRN*, *RECQL1*, *RECQL4*, and *RECQL5*, *BLM* forms the RecQ family of DNA helicases, a group of proteins that hydrolyse ATP to promote DNA unwinding [54]. Given the pivotal role of the RecQ family in various aspects of nucleic acid metabolism, it is logical that a deleterious phenotype in one of these genes could significantly contribute to cancer development. Indeed, individuals with syndromes associated with the lack of functional RecQ helicase proteins exhibit an increased predisposition to several types of cancer, including tumours of the breast, colon, lung, skin, or hematologic diseases (mainly leukaemias and lymphomas) [26]. The skin cancer predisposition in BS individuals (so far, most studies are in EUR ancestry), particularly basal cell carcinoma and squamous cell carcinoma, suggests a stronger susceptibility to ultraviolet (UV) radiation, despite some inconsistent evidence [52]. SSA populations are exposed to high levels of UV radiation [55], but dark skin confers natural protection against UV hazards, namely protection against degradation of folate [56]. It is unknown whether a BS patient of SSA ancestry would present with the skin rash symptom. Besides *BLM*, the interacting genes *RMI1*, *RMI2* and *TOP3A* had a higher essentiality in SSA than EUR TNBC cell lines (although not statistically significant after multiple-testing correction, probably due to the low available number of SSA TNBC cell lines). The same tendency in all these and other genes to be more essential in SSA ancestry rendered pathways related to DNA repair and replication to feature prominently for the SSA ancestry in the pathway enrichment analysis for gene essentiality. Also enriched in SSA essentiality were pathways related with ubiquitination and neddylation, PTMs of proteins coded by *BLM* and related genes, that we will discuss below in more detail.

Interestingly, the connection between cancer susceptibility and impaired DNA repair mechanisms was first established through the observation of CIN in cells of BS patients [57], and, as such, the crucial role of *BLM* as a genome gatekeeper has been extensively demonstrated since then. Particularly, BS individuals exhibit a wide range of genomic abnormalities, including elevated rates of mitotic recombination [58], sister chromatid exchanges (SCEs) [59,60,61], and chromosomal aberrations [62,63]. Additionally, these patients display impaired DNA replication processes, as evidenced by slower rates of DNA synthesis [64] and the accumulation of abnormalities during DNA replication [65]. In *BLM*-knockout mouse models, mammary tumours also exhibited a high frequency of SCEs and CIN [66]. Curiously, in the opposite direction, it has been shown, and we confirmed in this study, that *BLM* expression increases significantly in tumours in relation to normal tissues, indicating also a potential oncogenic role [53]. Moreover, overexpression of *BLM* has been associated with a lower overall survival time in lung and gastric cancer [67], as well as in myeloid leukemia and multiple myeloma [68,69], and prostate and colorectal cancer [70,71]. Regarding BC, a clinicopathological comprehensive study revealed a significant association between high *BLM* levels and more aggressive tumour phenotypes [34], namely with high histologic grade, larger tumour size, ER negativity, PR negativity, and TN phenotype. Through the analysis of TCGA RNA-Seq data, we showed increased expression of *BLM* in tumour tissues compared to their normal counterparts across nearly all cancer types, and TNBC is among the top ones. This aligns with previous findings [72], namely in hematological cancers [73], where tyrosine kinase fusion genes resulting from rearrangements have been linked to the induction of *BLM* expression [74]. For TNBC cohorts, both TCGA patients and cell lines, SSA-affiliated samples seem to have a tendency for low *BLM* gene expression, while all cases of high expression are of EUR ancestry. For the cancer cell line cohort, an identical trend for lower levels in SSA compared with EUR is observed for BLM protein expression. High *BLM* expressing EUR samples presented high numbers of SVs (driving a significant positive correlation). But some SSA and EUR low *BLM* expressing samples also presented high numbers of SVs, and this heterogeneity seems to indicate that other mechanisms of DNA repair are at play. Checking for *BRCA1*/*BRCA2* mutations in these cell lines, we found that an additional mechanism cannot explain that heterogeneity. So far, we do not know how to explain the seemingly lower mean *BLM* expression in SSA compared with EUR ancestry (we could not identify any possible expression quantitative trait locus in the expression data from the 1000 Genomes database).

Independently, we also recurred to the GDSC drug sensitivity data to address significant heterogeneity between the two ancestries for TNBC. The only significant result, after multiple-testing correction for 171 drugs, was observed for Pevonedistat, with SSA TNBC cell lines being more sensitive than EUR. This Pevonedistat finding was validated in vitro by us, using a subset of three SSA and three EUR TNBC cell lines. Pevonedistat is a potent and selective inhibitor of the NAE heterodimer, comprising NAE1 and UBA3 subunits, which is responsible for the initial and activating step of neddylation, a PTM process [75]. Similar to ubiquitination, neddylation enables the reversible conjugation of a factor (in the latter case, NEDD8) to substrate proteins. Ubiquitination primarily targets substrates for degradation in the proteasome, while neddylation modifies proteins by altering their properties, including conformational structure, stability, interactions with partners, or subcellular localization [76]. The most well-characterized substrates of neddylation are cullin-RING ubiquitin ligases (CRLs) [75], which regulate key biological processes, including cell cycle progression, gene transcription, apoptosis, signal transduction, or DNA replication, among others [77,78]. Non-cullin proteins are also targeted by neddylation, including tumour suppressors (such as *TP53*, *PTEN* and *PARP1*) and oncogenes (*HER2*, *AKT* and *EGFR*) [79]. So, dysregulation of this system (overactivation) may pathologically result in cancer development [80], rendering neddylation an interesting target for cancer treatment [81].

Extensive preclinical evidence has demonstrated Pevonedistat’s potential as an effective anti-cancer agent, alone or in combination, across various cancer cell types, including BC [82,83,84,85] and TNBC [85,86,87,88]. A NEDD8 enrichment was observed in TNBC cells, rendering them particularly vulnerable to NEDD8 depletion [89]. These promising findings led to the inclusion of Pevonedistat in more than 40 phase I-III clinical trials (as registered in https://www.clinicaltrials.gov/, accessed on 13 January 2025), primarily for hematological malignancies and, to a lesser extent, solid tumours [90,91,92,93,94]. Published results to date suggest an efficacy profile impacting prolonged stable disease, partial response, and complete response [95]. Pevonedistat is generally well-tolerated in mice [79]. However, in humans, numerous clinical trials have reported side effects, including dose-limiting toxicities such as arthralgia, atrial fibrillation, bone pain, diarrhea, hypertension, mediastinal hemorrhage, febrile neutropenia, elevated liver enzymes, muscle cramps, and thrombocytopenia [91,96]. The Pevonedistat concentration used in this study for the EUR TNBC cell lines was relatively high compared with concentrations previously reported in in vitro TNBC studies [88,97]. In Powell et al. [88], Pevonedistat was tested at 0.1, 1, and 10 μM, with cells exposed to the drug for 72 h. In another study, Pevonedistat was evaluated across a concentration range of 10–1000 nM, with an exposure period of 84 h. In addition, our analysis of the GDSC dataset yielded an estimated average IC_50_ of 21.5 μM for the EUR TNBC cell lines, based on a 72 h exposure. The higher concentration used in our study may, at least in part, reflect the shorter exposure period of 48 h, as a higher drug concentration may have been required to achieve a comparable degree of growth inhibition. We also confirmed in the GDSC drug sensitivity data that SSA TNBC cell lines were more sensitive than EUR to the cisplatin drug, although not statistically significant after multiple-testing correction. Cisplatin, a first-generation platinum agent, is widely used in the clinical setting for cancer treatment, including TNBC [98], although the response to this drug class is conditioned by the molecular heterogeneity of this BC subtype [99].

Interestingly, we were able to establish a link between the two independent tests we performed in this work, demonstrating higher essentiality of *BLM* and sensitivity to Pevonedistat in SSA compared to EUR TNBC cases. Our transcriptomic analysis of SSA and EUR TNBC cell lines exposed to the respective maximum concentration of Pevonedistat identified a significant downregulation of *BLM*-centred (and other) DNA damage repair pathways. A hypothesis is that the drug acting on the CRLs will slightly but synergistically reduce the activity of several helicases (including *BLM*, *WRN*, *RECQL4*) and associated genes. This may indicate that the regulatory mechanism involving *BLM* is probably more complex and could involve feedback mechanisms, and this should be tested across more models. To date, there is no evidence of neddylation of the BLM protein, but several PTMs (phosphorylation, ubiquitination and SUMOylation) tightly control BLM activity, levels during the cell cycle, response to replicative stress, localization or fidelity of chromosome segregation [100]. A pharmacotranscriptomics study on patient-derived xenograft models further highlights the interplay between PTMs in the context of Pevonedistat activity [88]. The authors showed that there is a combined action of neddylation and SUMOylation pathways in Pevonedistat activity, and that levels of NEDD8 and SUMO1 were determined to be positively correlated with the drug’s response. Further functional investigation is needed.

## 4. Materials and Methods

### 4.1. Ancestry Information for Cancer Cell Lines and TCGA Patients

We used the ancestry inference from Dutil et al. (2019) for the cancer cell lines from the Catalogue of Somatic Mutations in Cancer (COSMIC) and CCLE panels [24]. As most of the proxies for SSA ancestry are admixed African Americans (AA), we considered a cell line as SSA if the African component was above the reference admixture values described by Patin et al. (2017) [51] in AA: 70% African admixed with ~30% European/Native American background.

Ancestry inferences for TCGA BC samples and the TNBC subset were pulled from Carrot-Zhang et al. (2020) [17]. We excluded samples without consensus ancestry categorization; not classified as African or EUR; and classified as African or EUR in the consensus calls, but whose admixture percentages for those clusters were less than 70%.

### 4.2. Cancer Cell Lines Gene Dependency, Drug Sensitivity and Multiomics Data

Cancer cell lines gene-dependency data, published by the Achilles (Broad Institute, Cambridge, MA, USA; [101,102]) and SCORE (Sanger Institute, Hinxton, UK; [6]) projects, and available at the DepMap Portal (22Q2 DepMap Public release; https://depmap.org/portal/, accessed on 21 September 2022) were used for the gene essentiality analyses. Annotation files from those institutional public repositories (Broad’s DepMap portal: https://depmap.org/portal/, accessed on 21 September 2022; Sanger’s CMP portal: https://cellmodelpassports.sanger.ac.uk/, accessed on 21 September 2022) were mined for tissue of origin, cancer type, growth properties, gender and barcode harmonization, while cancer subtype of the cell lines, specifically TNBC status, was checked in the Cellosaurus resource (v.42.0) [103]. The 22Q2 DepMap gene-dependency dataset contains 17,386 gene-dependency profiles across 1086 cell lines, and results from the integration of gene effect scores derived from CRISPR-Cas9 screens performed by both institutes [5]. Negative values are indicative of a detrimental impact of a knockout gene on cell survival. The generated joint dataset was scaled to ensure that the median score of nonessential genes rounds to 0 and that of essential pan-cancer genes equals −1.

Only 29 cancer cell lines included in the Achilles and SCORE studies were TNBC. We subsequently filtered the gene effect matrix obtained from CRISPR screen data for these 29 cell lines, specifically selecting those exhibiting an adherent growth pattern and having an SSA or EUR ancestral background. We ended up with a final selection of 16 TNBC cell lines for the gene-dependency investigation: four SSA (HCC1806, MDA-MB-468, MDA-MB-157, HCC70) and 12 EUR (HCC1143, Hs578T, BT-549, MDA-MB-231, MDA-MB-453, MFM-223, HCC38, HCC1937, CAL-120, MDA-MB-436, HCC1395, CAL-51). For the sake of consistency, in all subsequent analyses, we only considered this set of adherent TNBC cell lines.

Drug sensitivity data in cancer cell lines were obtained from the GDSC Project repository (https://www.cancerrxgene.org/, accessed on 20 January 2023; Release 8.4—dataset GDSC2) [104]. For evaluation of TNBC ancestry influence in drug sensitivity, the dataset comprised 18 TNBC cell lines: four SSA (HCC1806, MDA-MB-468, MDA-MB-157, HCC70) and 14 EUR (HCC38, HCC1143, HCC1395, HCC1937, Hs578T, BT-20, BT-549, MDA-MB-231, MDA-MB-453, MFM-223, CAL-51, CAL-120, MDA-MB-436, HDQ-P1). Additional information on the “BRAF_mut (BRAF^V600E^)” status to be considered on the GDSC2 drug sensitivity matrix was kindly provided by Francesco Iorio, based on data published in Iorio et al. (2016) [7].

Inferred data on mRNA levels (RNA-Seq), protein abundance (DIA-MS), somatic mutations (SMs) (whole exome sequencing [WES]), copy number variants (CNVs) (WES), and SVs (RNA-Seq) were downloaded from the CMP portal (https://cellmodelpassports.sanger.ac.uk/, accessed on 27 January 2023).

### 4.3. TCGA Cancer Patient Datasets

TCGA TNBC cases were identified after careful inspection of clinical data of patients included in the BC TCGA cohort (named as BRCA), as having negative status of ER, PR and HER2 receptors, totalling 116 cases. From these, 25 were SSA and 64 EUR (as three EUR cases had MSI; information from [105]). We then queried the Genomic Data Commons Data Portal (https://portal.gdc.cancer.gov/, accessed on 18 April 2023) for SMs (*n* = 77, WES), SVs (Arriba and STAR-Fusion pipelines; *n* = 89, RNA-Seq), and gene expression (*n* = 89, RNA-Seq in TPM). The somatic mutational burden was estimated as the ratio between the number of SMs per sample and the total number of sequenced bases in the WES (~38 Megabases; [106]). Copy number variation data were retrieved from the COSMIC (https://cancer.sanger.ac.uk/cosmic; release 98, accessed on 18 April 2023) database. Ploidy and purity were reported elsewhere [107,108,109]. Curated and filtered TCGA follow-up data were retrieved from Liu et al. (2019) [110].

### 4.4. Confirmation of Identity and Ancestry Affiliation of TNBC Cell Lines

To confirm the identity of the TNBC cell lines that we used in the lab experiments, for the lines acquired for this work (from ATCC—HCC1806, HCC70, HCC1395, MDA-MB-436 and MDA-MB-231) and for the ones already available at i3S cancer cell lines biobank (Hs578T, BT-20 and MDA-MB-468), we genotyped 15 STRs + Amelogenin loci, included in CLA IdentiFiler™ Plus PCR Amplification Kit (Thermo Fisher Scientific, Waltham, MA, USA), run in a 3500xL Dx Genetic Analyzer (Thermo Fisher Scientific, Waltham, MA, USA).

To confirm the ancestry affiliation of the TNBC cell lines, we genotyped > 850,000 variants included in the Axiom™ Precision Medicine Diversity Array (PMDA, Thermo Fisher Scientific, Waltham, MA, USA), which was genotyped in the GeneTitan MC Fast Scan Instrument (Thermo Fisher Scientific, Waltham, MA, USA). The software ADMIXTURE (v.1.3.0) [111] was applied to the profiles of TNBC cell lines after merging with a panel of worldwide populations from the 1000 Genomes project (on GRCh38) [48,112,113] reference after pruning for linkage disequilibrium, by removing any single nucleotide polymorphism (SNP) that had an r^2^ > 0.2 with another SNP, within a 50-SNPs sliding window with steps of 5 SNPs. We limited ADMIXTURE using maximum likelihood for components (K) equal to 3.

### 4.5. Evaluation of Chromosome Instability and Immunogenicity

Micronuclei occurrence and cGAS (a cytosolic DNA sensor) were assessed in the two SSA (HCC1806 and MDA-MB-468) and three EUR (MDA-MB-231, BT-20 and Hs578T) TNBC cell lines by immunofluorescence. Two conditions were tested: normal cell division and after arresting cell division at the G2/M boundary with the drug RO-3306 (Selleck Chemicals LLC, Houston, TX, USA). Briefly, TNBC cells were seeded on 24-well plates and, after 24 h for adhesion, were exposed for an additional 24 h to RO-3306 (10–15 µM). A portion of these cells was followed for cytometry analysis based on propidium iodide staining on a BD Accuri C6 Plus (BD Biosciences, Franklin Lakes, NJ, USA), excited at 488 nm, to check how many cells were in different cell division phases. Another portion was used for the immunofluorescence, for which cells were fixed with 4% paraformaldehyde in PBS for 20 min. Following fixation, cells were rinsed in PBS, permeabilized in PBS + 0.3% Triton X-100 for 7 min, and then blocked in 10% FBS + PBS for 1 h. Both anti-cGAS primary (mouse anti-cGAS 1:250; #sc-515777, Santa Cruz Biotechnology, Dallas, TX, USA) and secondary (^m^Alexa488, 1:400; #A32723, Thermo Fisher Scientific, Waltham, MA, USA) antibodies were diluted in PBS + 0.05% Tween-20 containing 5% FBS. DNA was counterstained with DAPI (1:4000; D9542, Sigma) for 10 min. Fixed cells were imaged using a motorized inverted fluorescence microscope, Leica DMI6000 FFW (Leica Microsystems, Wetzlar, Germany). Fixed-cell experiments (micronuclei counts and cGAS positivity) were quantified using ImageJ/Fiji software (v. 2.14.0).

### 4.6. In Vitro Drug Test and RNA-Seq Analysis

In vitro drug assays with Pevonedistat were conducted in three SSA (HCC1806, HCC70 and MDA-MB-468) and three EUR (BT-20, HCC1395, and MDA-MB-436) TNBC adherent cell lines. The cells were seeded in medium supplemented with 5% FBS, and after 24 h (when they reached 70% confluency), Pevonedistat was independently added in two-fold serial dilutions of 200 µM for 48 h. Dimethyl sulfoxide (DMSO) was used as the vehicle control. The GI_50_ (drug concentration that inhibits 50% of the cell growth) of Pevodenistat in each cell line was determined by the sulforhodamine B assay, following the published protocol [114]. For each cell line, three independent experimental replicates (*n* = 3) were performed, with the GI_50_ value reported as the mean of the three replicate-specific GI_50_ estimates and represented graphically as dose–response curves.

In order to characterize the molecular impact of the drug through transcriptomics, we repeated the experiment, but added Pevonedistat at the average GI_50_ values for each ancestral group: [Higher] 78.98 µM for EUR; [Lower] 0.72 µM for SSA. At a 48 h time point, both treated and control cells were harvested and preserved in RNAlater RNA Stabilization Solution (Thermo Fisher Scientific, Waltham, MA, USA) for full transcriptomic characterization. RNA libraries were generated through polyA capture (or rRNA removal) and reverse transcription of cDNA, which were then sequenced as 150 bp paired-end reads on an Illumina NovaSeq 6000 System (Illumina, San Diego, CA, USA). Raw reads were aligned against the human reference genome GRCh38 resorting to the STAR aligner, and the count of reads attributed to genomic features for each sample was determined utilizing the R library featureCounts.

### 4.7. Differential Essentiality Analysis

For the differential gene essentiality evaluation, we recurred to the R/Bioconductor package limma (v.3.54.2) [115], applying a linear model to the matrix of gene effect scores on the four SSA and 12 EUR adherent TNBC cell lines. The analysis operated on the explanatory variable “ancestry”, using “SSA” as the reference. We tested the model with and without the intercept term (as explained in [116]), confirming both gave the same results. Multiple-testing adjustment was performed using the Benjamini–Hochberg method. For the validation of this methodology, the same model was applied to GDSC drug sensitivity data (natural logarithm [ln] of IC_50_ values) on the cell line *BRAF* mutational status.

To assess the robustness of the differential essentiality findings, a LOO analysis was performed. Briefly, the original differential gene essentiality model was iteratively repeated, with one cell line excluded from the dataset while all remaining cell lines were retained.

### 4.8. Group-Wise Comparison Analyses of Multiomics Data and Drug Sensitivity

The group-wise comparisons were statistically evaluated through the two-sided Wilcoxon rank-sum test for the cancer cell line data, and by the two-sided unpaired t-test for TCGA data. Boxplots and statistical tests were generated in RStudio (v.2024.09.0 + 375).

### 4.9. Gene Set Enrichment Analyses

To identify enrichment of gene sets in SSA and EUR TNBC cell lines, TCGA samples and RNA-Seq data obtained here, the respective normalized protein-coding gene expression matrices were inputted in the GSEA (v.4.1.0) [117], using GO-BP, GO-CC, and GO-MF as gene sets. We conducted GSEA pre-ranked analyses, using the tool’s default parameters, and SSA ancestry as the reference group (positive normalized enrichment score [NES] values).

ssGSEA was implemented on the results obtained in the differential essentiality analysis in SSA vs. EUR TNBC cell lines. For this, ordered (descending) logFC of gene effect scores from limma output were used as input in ssGSEA2.0 in the R environment [118]. The GO-BP, GO-MF and GO-CC gene sets were used in both analyses, with default parameters.

Top hits for the enrichment results were plotted as flipped bar plots in the R library ggplot2 (v.3.4.2).

### 4.10. Correlation Between BLM Expression and Somatic Mutational Profiles

Spearman pairwise correlations were computed between *BLM* expression and somatic mutational profiles (SM, CNVs and SVs; ln-transformed), in TNBC cell lines and TCGA samples, respectively. Data were analyzed in Python 3.10.6, utilizing pandas (v.2.0.1), scipy (v.1.10.1) and numpy (v. 1.24.3) modules, and graphically represented by heatmaps with seaborn (0.12.2) and matplotlib (3.7.1) modules.

### 4.11. Evaluation of Differential Drug Sensitivity

The GDSC2 dataset included the test of 171 drug sensitivities (as ln of IC_50_ values) in the four SSA versus 14 EUR TNBC cell lines (only drugs with values for all these cancer cell lines were considered). The differential drug sensitivity between the two ancestries was tested with the limma linear model, as for differential gene essentiality analysis described before, as well as a LOO analysis.

## 5. Conclusions

Taken together, the results of this work are relevant to exploring novel mechanisms underlying TNBC development and treatment in the SSA context. At least in vitro, Pevonedistat exhibited differential sensitivity in TNBC SSA ancestry cell lines, at significantly lower doses than in EUR counterparts. Further investigation is needed to understand in detail the molecular action of Pevonedistat on the pathways related to DNA damage repair, in which *BLM* plays a main role. This could uncover novel and specific pathways and targets, thereby facilitating the development of more directed treatment strategies. If the limited number of SSA TNBC cell lines included here may hinder the identification of stronger associations or even novel ones, our results clearly support the need to establish more in vitro cancer models for SSA ancestry, as significant and informative differences exist between ancestries.

## Data Availability

The transcriptomic dataset generated during the current study is available in the European Nucleotide Archive (ENA) repository at PRJEB113435.

## Supplementary Materials

The following supporting information can be downloaded at https://www.mdpi.com/article/10.3390/ijms27219980/s1.

## Abbreviations

The following abbreviations are used in this manuscript:

BC	Breast cancer
BLM	BLM RecQ-like helicase
BP	Biological Processes
BRAF	B-Raf proto-oncogene
BS	Bloom syndrome
CC	Cellular Components
CCLE	Cancer Cell Line Encyclopedia
CIN	Chromosomal instability
CMP	Cell Model Passports
CNVs	Copy number variants
COSMIC	Catalogue of Somatic Mutations in Cancer
CRLs	Cullin-RING ubiquitin ligases
ER	Estrogen receptor
EUR	European
GDSC	Genomics of Drug Sensitivity in Cancer
GO	Gene Ontology
GSEA	Gene Set Enrichment Analysis
HR	Homologous recombination
ln	Natural logarithm
logFC	Log2-fold changes
MF	Molecular Function
MSI	Microsatellite instability
NAE	Nedd8 activating enzyme
NES	Normalized enrichment score
PR	Progesterone receptor
PTMs	Post-translational modifications
RMI1	RecQ-mediated instability protein 1
RMI2	RecQ-mediated instability protein 2
SCEs	Sister chromatid exchanges
SMs	Somatic mutations
SSA	Sub-Saharan African
ssGSEA	Single-sample GSEA
STRs	Short Tandem Repeats
SUMOs	Small ubiquitin-related modifiers
SVs	Structural variants
TCGA	The Cancer Genome Atlas
TNBC	Triple-negative breast cancer
TOP3A	Topoisomerase IIIa
TPMs	Transcript per million
UV	Ultraviolet
WES	Whole exome sequencing
