# Supplementary material for: In Vitro Models of Triple-Negative Breast Cancer of Sub-Saharan African Ancestry Suggest BLM-Centred DNA Damage Repair Dependency and Pevonedistat Sensitivity
Source: Int J Mol Sci. 2026 Nov 7;27(21):9980. doi: 10.3390/ijms27219980 (PMC13764445; doi:10.3390/ijms27219980)
Supplement: Supplementary file 1 [file ijms-27-09980-s001.zip › Supplementary Figures.pdf]

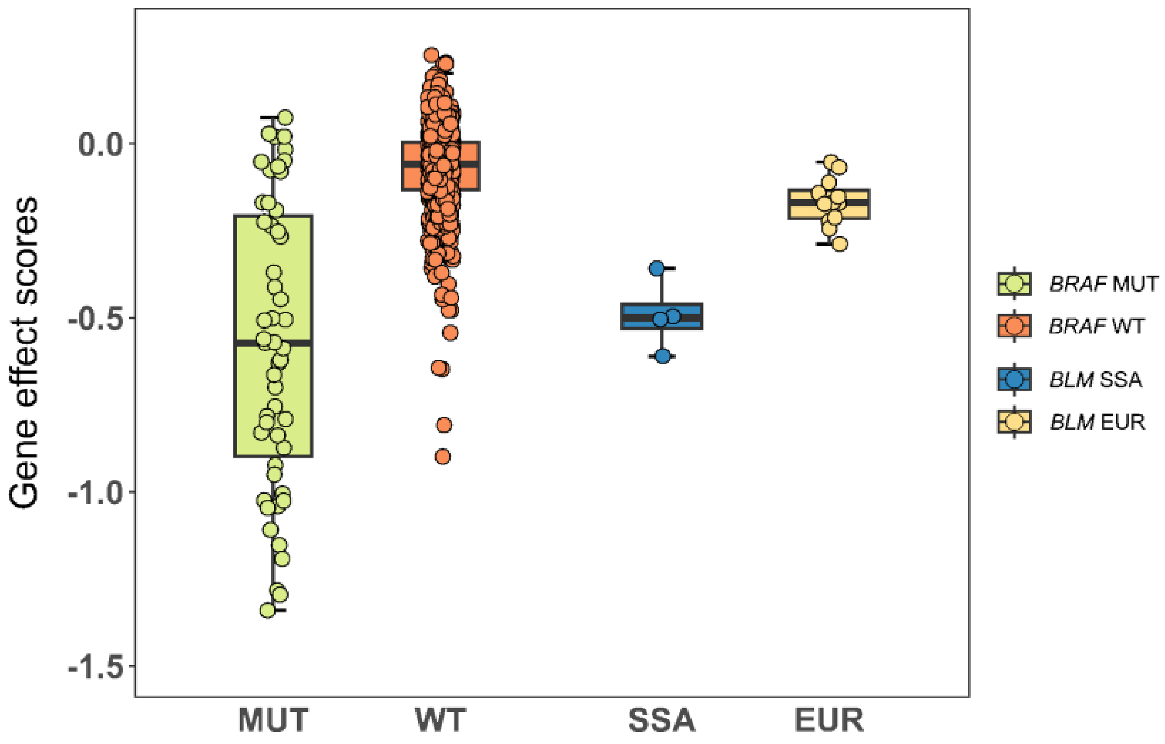

Figure S1 – Comparison of dependency between *BLM*-ancestry and BRAFV600E gain-of-function mutation (MUT; n=51) versus wild type (WT; n=535).

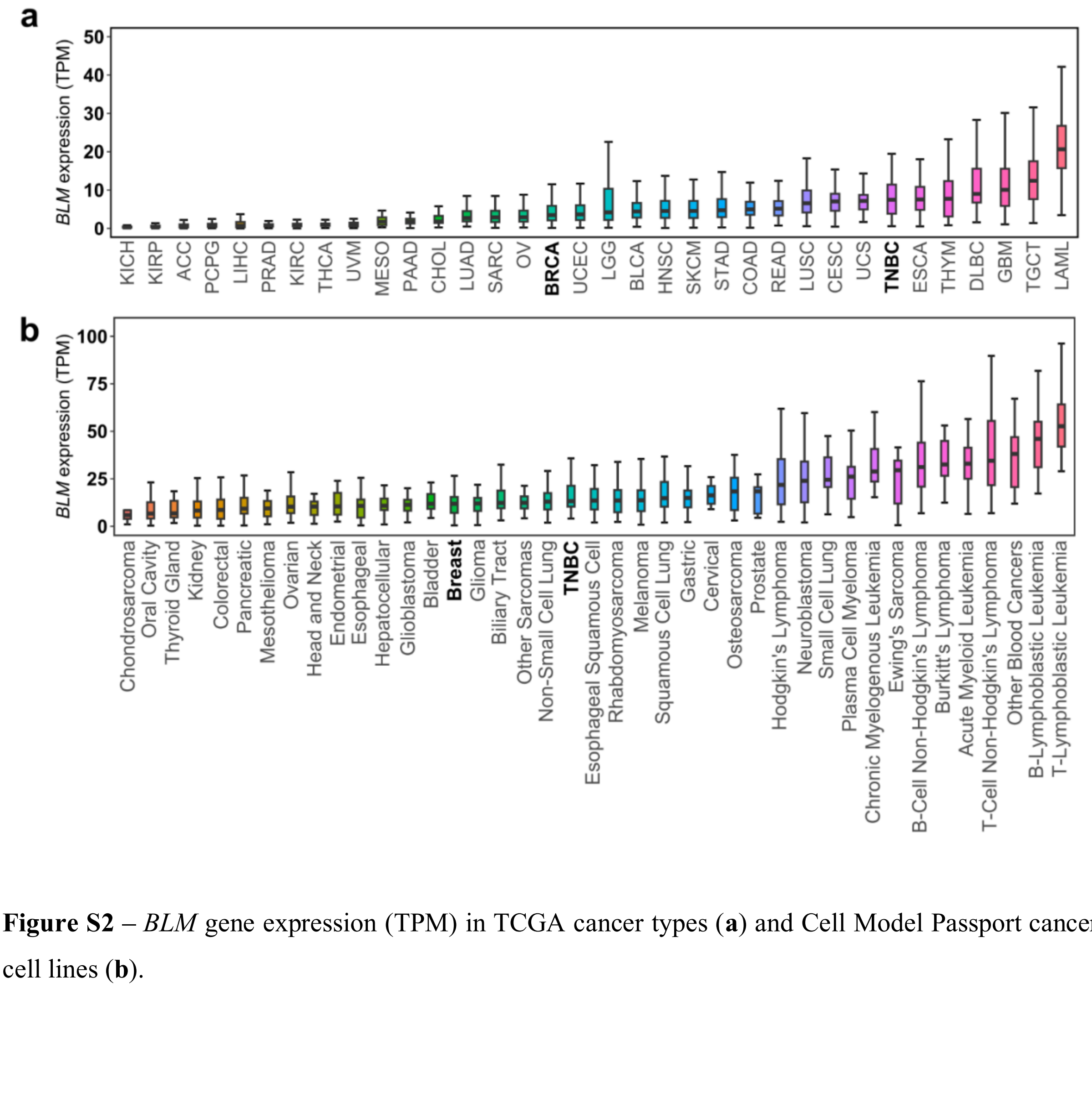

Figure S2 – *BLM* gene expression (TPM) in TCGA cancer types (**a**) and Cell Model Passport cancer cell lines (**b**).

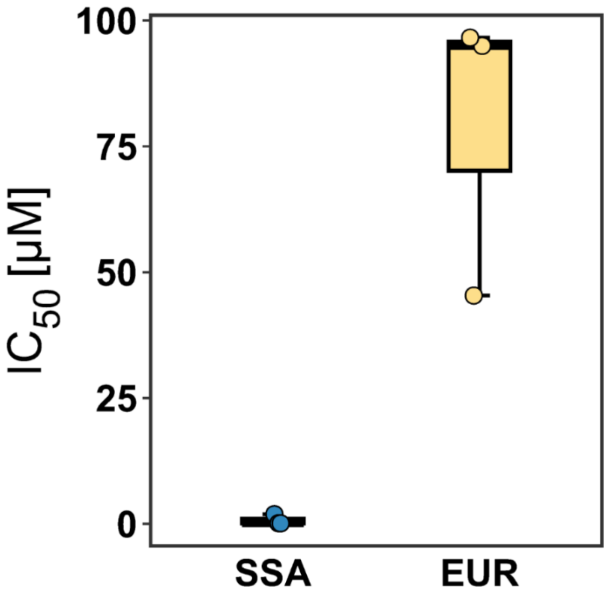

Figure S3 – GI_{50} values for the *in vitro* assay with Pevonedistat conducted in this work.

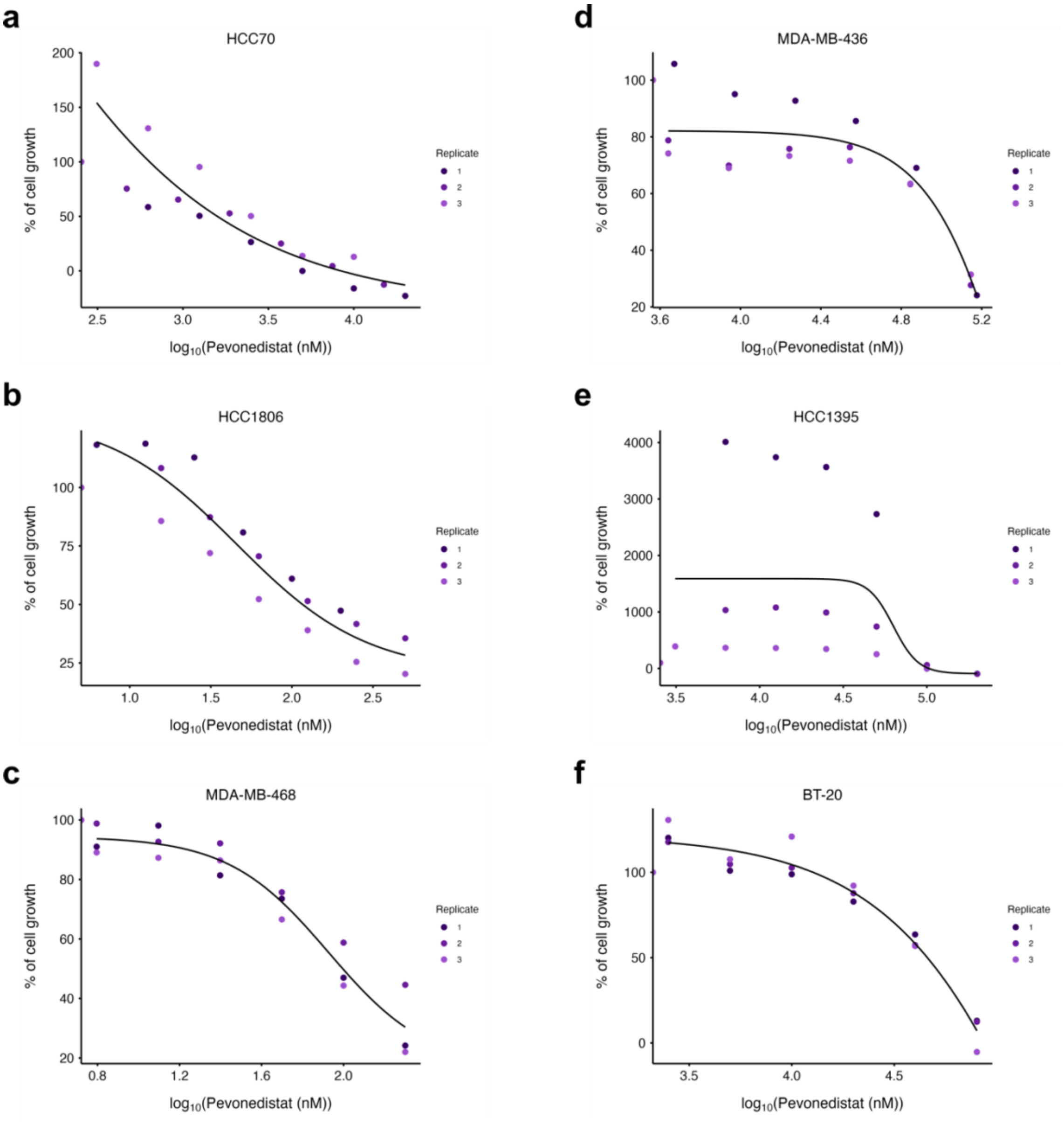

Figure S4 - Dose–response profiles of TNBC cell lines included in the pharmacotranscriptomics assay. Dose–response profiles are shown for three SSA cell lines, HCC70 (**a**), HCC1806 (**b**), and MDA-MB-468 (**c**), and three EUR cell lines, MDA-MB-436 (**d**), HCC1395 (**e**), and BT-20 (**f**). Individual points represent measurements from independent experimental replicates, with colours indicating the respective replicate.

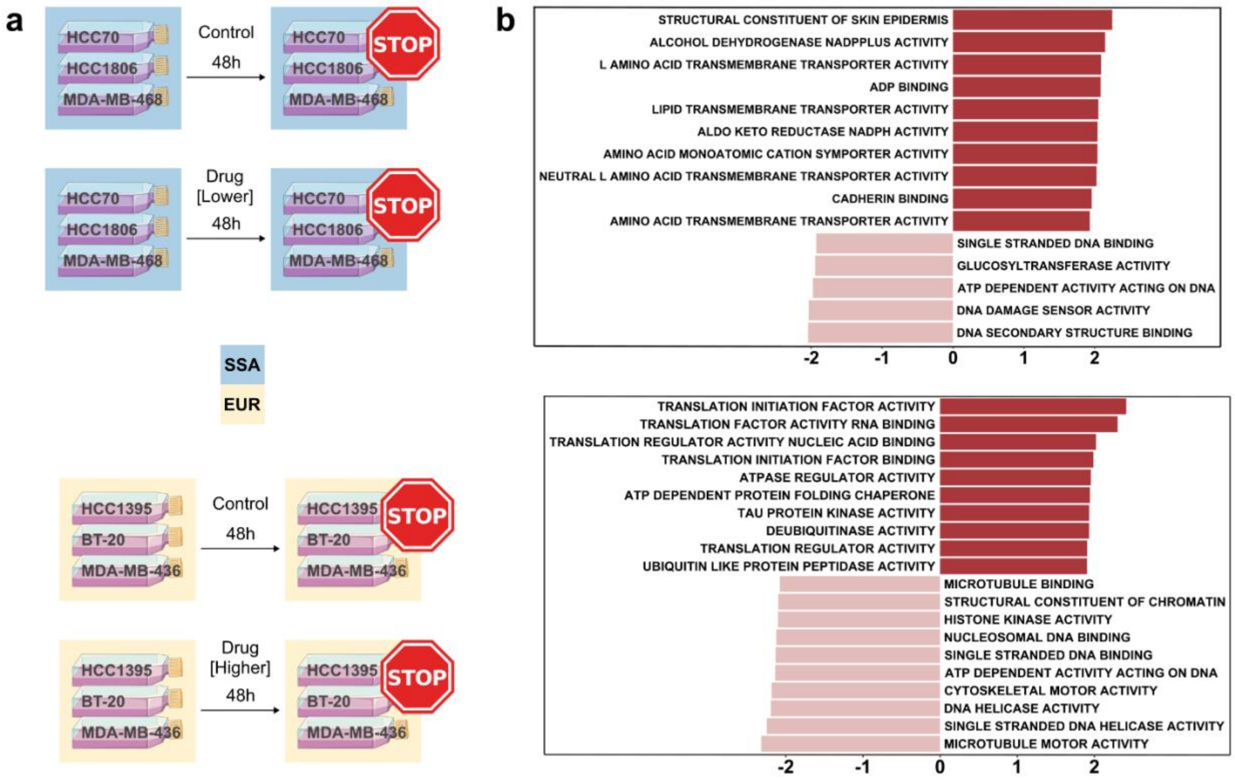

Figure S5 - a. *In vitro* test of Pevonedistat in three SSA and EUR TNBC cancer cell lines, at the respective median GI_{50} concentrations for each ancestral group ([Higher] of 78.98 µM for EUR; [Lower] 0.72 µM for SSA). b. Top10 enrichment results (GO-MF) for the RNASeq data on the *in vitro* test of Pevonedistat between SSA and EUR TNBC cancer cell lines; raw data is available in Tables S18 and S19, respectively.
